# Caught in Transit: Thrombus Traversing a Patent Foramen Ovale

**DOI:** 10.7759/cureus.87048

**Published:** 2025-06-30

**Authors:** Sri Nuvvula, Tanzim Bhuiya, Ji-Cheng Hsieh, Joshua P Gilman, John N Makaryus

**Affiliations:** 1 Department of Internal Medicine, Donald and Barbara Zucker School of Medicine at Hofstra/Northwell, Manhasset, USA; 2 Department of Internal Medicine, Hospital of the University of Pennsylvania, Philadelphia, USA; 3 Department of Cardiology, Northwell Cardiovascular Institute, Manhasset, USA

**Keywords:** patent foramen ovale, pulmonary embolism (pe), stroke, thrombus in transit, transesophageal echocardiography

## Abstract

While a patent foramen ovale (PFO) is a very common anatomic variant, thrombus-in-transit through a PFO is a rare and potentially life-threatening event. Such thrombi can lead to paradoxical emboli, causing multi-organ thromboembolic events with significant hemodynamic and functional consequences depending on the affected systems. Here, we present a case of a 65-year-old man with hypertension who arrived with neurologic deficits, found to have an acute left temporal lobe infarction, and was subsequently found to have a saddle pulmonary embolism. Transesophageal echocardiography then revealed a ten-centimeter thrombus-in-transit across a PFO, and he was later found to have a deep vein thrombosis. The PFO was determined to be the source of his systemic and pulmonary emboli. The patient, initially ineligible for thrombolytics, was managed with mechanical thrombectomy and systemic anticoagulation, leading to gradual clinical improvement.

The patient stabilized post-thrombectomy and was ultimately discharged. On ambulatory follow-up, the patient was asymptomatic, without further clotting events. Repeat anticardiolipin testing was negative. This case illustrates the critical need for urgent anatomical assessment for PFO in patients presenting with concurrent right- and left-sided thrombi suggestive of paradoxical embolism. Early detection of thrombus-in-transit can guide risk stratification and inform management, which could potentially improve outcomes for patients with complex thromboembolic presentations.

## Introduction

A patent foramen ovale (PFO) is a common anatomic abnormality that connects the pulmonary and systemic circuits. Paradoxical embolism can occur in the setting of a PFO, where a venous thrombus crosses to the systemic circulation, potentially leading to complications such as stroke, pulmonary embolism (PE), acute mesenteric ischemia, or acute limb ischemia. Specifically, ischemic stroke has been shown to be approximately four times more frequent in patients with PFO [1]. Large thrombi are rarely visualized on diagnostic imaging as a ‘thrombus in transit’ (TIT), a thrombus moving within the heart, seen in <4% of PE cases [2]. There are rare reports in the literature of patients with a TIT through a PFO, often noted on computed tomographic (CT) or echocardiographic imaging in the setting of concomitant PE [3-6]. PE with concomitant TIT is not common, with an incidence as low as 3.8% of observed PE cases, and is associated with significantly higher early mortality when treated with anticoagulation alone [2]. These cases typically involve arterial occlusion in one or two organ systems, without diffuse thromboembolism [3-6].

While anticoagulation is a mainstay of treatment in thromboembolic events, prior cases of PE with imaging findings noting a TIT across a PFO required interventions including thrombolytics, embolectomy, or thrombectomy for definitive management of a significant thromboembolic burden [3-5]. We describe a novel case of a patient who presented with a stroke and was then found to have a saddle PE. Subsequently, a TIT through a PFO was discovered on echocardiographic imaging, likely originating from deep venous thrombosis and patient required a mechanical thrombectomy and a PFO closure. Our case is distinct in presenting a patient with multi-organ embolization, unlike what has been reported in the literature [3-6], highlighting the importance of PFO evaluation in patients with echocardiographic findings concerning for elevated right-sided pressures from acute PE.

## Case presentation

**Figure 1:**
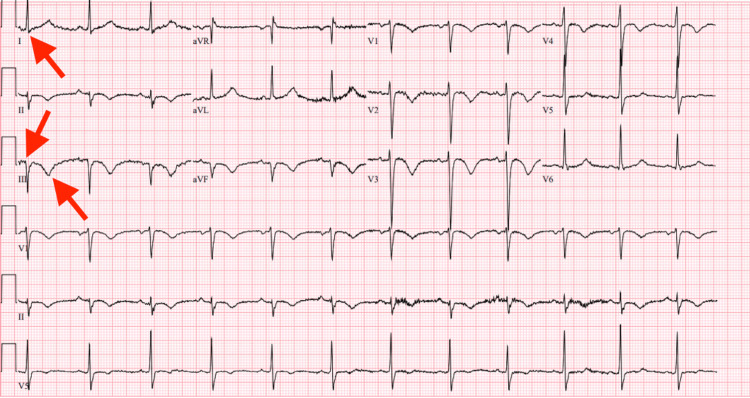
ECG with red arrow pointing to s-wave in lead I, and red arrows pointing to a q-wave and inverted t-wave in lead III.

A 65-year-old man with a past medical history of hypertension presented to the Emergency Department with acute facial droop and slurred speech that progressed to global aphasia. On admission, the patient’s vital signs were as follows: blood pressure 150/100 mmHg with a heart rate of 102 beats per minute, a respiratory rate of 16 breaths per minute, and oxygen saturation of 97% on room air. The physical exam revealed a regular heart rate and rhythm, non-labored respirations, no visible limb deformities, non-tender extremities, and no rashes or bruises. The National Institutes of Health Stroke Scale score was 9; the patient received 2 points for incorrectly answering their age and the month, 2 points for not opening and closing eyes or gripping a hand on command, 3 points for not having usable speech, and 2 points for having slurred speech that was unintelligible. His last known normal was 13 hours before presenting to the hospital. No other motor or sensory deficits were noted on physical exam. Initial laboratory work-up was notable for a prothrombin time (PT) of 15.3 sec (10.5-13.4 sec), International Normalized Ratio (INR) 1.27 (0.88-1.16), and partial prothrombin time (PTT) 36.0 sec (27.5-35.5 sec). Troponin I and N-terminal pro-B-type natriuretic peptide (pro-BNP) were elevated to 523 ng/L (ULN < 78.5 ng/L) and 5441pg/mL (0-125 pg/mL), respectively. Electrocardiogram (ECG) demonstrated sinus rhythm with an s-wave in lead I and both a q-wave and an inverted t-wave in lead III without any clear evidence of ischemia (Figure 1).

Imaging work-up obtained within an hour of presentation was notable for a CT of the head without contrast demonstrating an acute left temporal lobe infarct without evidence of acute intracranial hemorrhage or brain mass.

**Figure 2:**
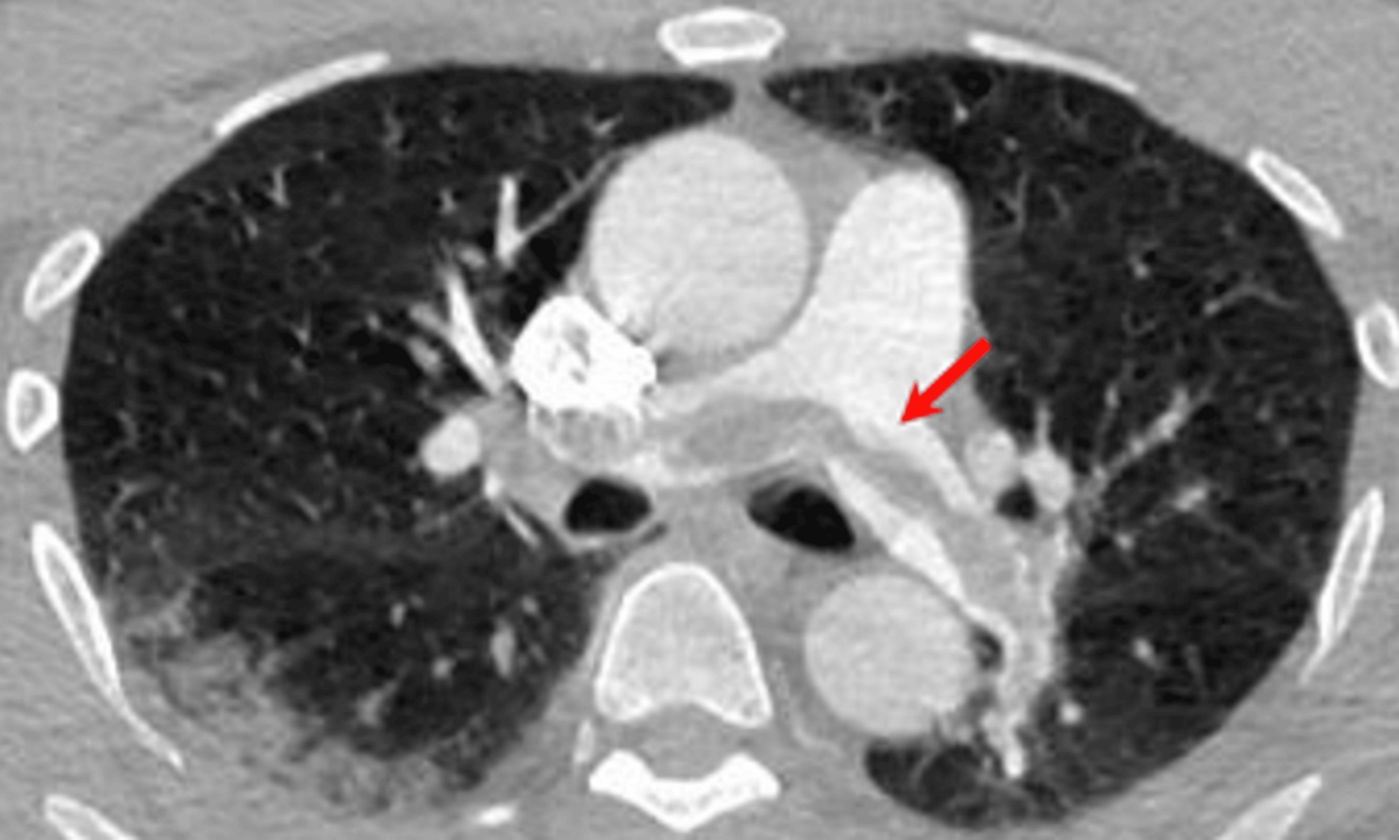
Saddle embolism in pulmonary artery seen on CTA neck which was contrast-enhanced with red arrow pointing to saddle embolism. This embolism required pulmonary embolectomy and anticoagulation to prevent likely clinical decompensation. CTA: computed tomography angiography.

CT angiography of the brain and neck demonstrated patent intracranial circulation, no flow-limiting stenosis or occlusion, and showed patent cervical vasculature without flow-limiting stenosis or occlusion but demonstrated saddle PE (Figure 2).

**Figure 3:**
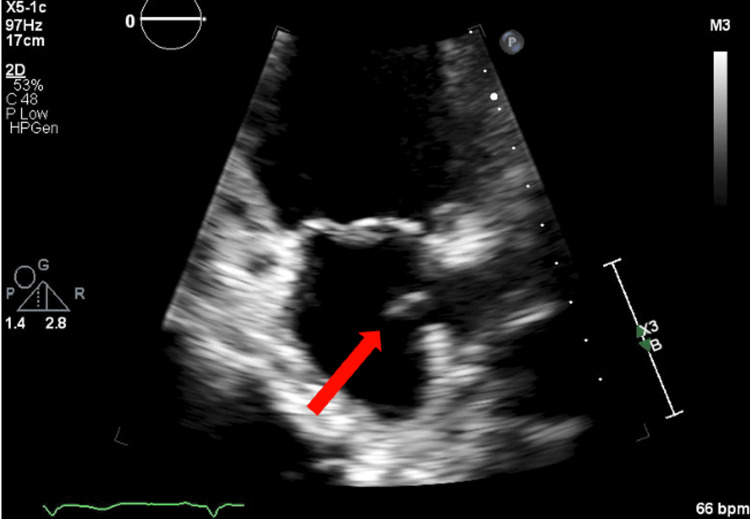
TTE in the apical right ventricle focused view, zoomed in on the right atrium showing a mobile echo-bright mass in the right atrium abutting the atrial septum with newly reduced EF of 40% and reduced right ventricular systolic function. TTE: transthoracic echocardiography; EF: ejection fraction.

Full-dose anticoagulation with heparin was initiated after determining that the benefit of anticoagulation in the setting of PE outweighed the risk of hemorrhagic transformation of the left temporal infarct. Transthoracic echocardiography (TTE) showed a thrombus with right to left ventricle ratio of 1:1. (Figure 3).

**Figure 4:**
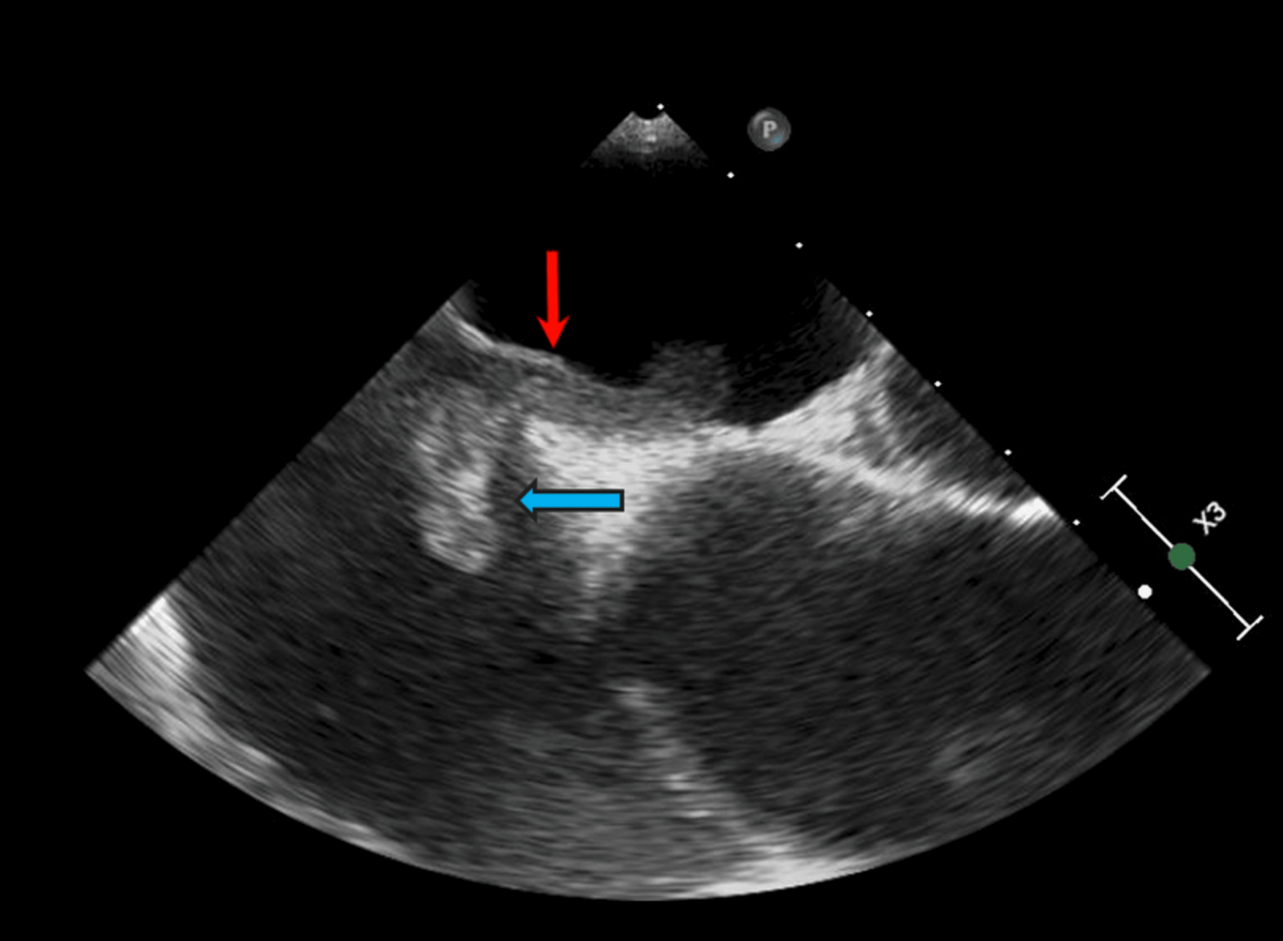
TEE in the mid-esophageal four chamber view showing a TIT measuring approximately ten centimeters in length across a PFO. The red arrow shows PFO and blue arrow shows the clot. This TIT required thrombectomy to prevent systemic embolism. TEE: transesophageal echocardiography; TIT: thrombus in transit; PFO: patent foramen ovale.

The patient was admitted to the Cardiac Intensive Care Unit for elevated clinical monitoring and urgent transesophageal echocardiography (TEE) 1 day after admission for consideration of thrombectomy. TEE revealed a mobile thrombus crossing the PFO, consistent with a TIT (Figure 4).

The imaging modalities utilized in the work-up of this patient and their pertinent findings are summarized in Table 1.

**Table 1:** Imaging utilized in the diagnostic work-up and their pertinent findings.

Imaging modality	Clinical finding
Electrocardiogram	S-waves in lead I and inverted t-waves and q-waves in lead III.
Computed tomography angiography neck	Saddle embolism in pulmonary artery.
Transthoracic echocardiogram	A mobile echo-bright mass in the right atrium abutting the atrial septum.
Transesophageal echocardiogram	A thrombus in transit across a patent foramen ovale.

A video of the clot in transit is seen on TEE in Video 1.

**Video 1:** A video of the clot in transit is seen on TEE. TEE: transesophageal echocardiography.

Subsequent lower extremity duplex ultrasonography showed a deep venous thrombosis (DVT) in the left lower extremity that was asymptomatic, determined to be the likely source of the TIT and the patient’s PE, and stroke. The patient was brought to the operating room two days after initial presentation for embolectomy given the TIT and the potential of recurrent systemic embolization. At operation, an approximately 2-inch, embolic thrombus was removed from across the PFO. No other intracardiac thrombus was identified. In addition, a pulmonary embolectomy was performed, removing clots from the right and left pulmonary arteries. The patient had no prior history of atrial fibrillation, but due to possible underlying atrial fibrillation as the etiology of the patient’s stroke, an AtriClip (AtriCure, Inc., Mason, OH, USA) was applied to occlude the left atrial appendage. Although AtriClip typically is not placed in the absence of documented atrial fibrillation, it was determined that the patient had a high risk of having atrial fibrillation, given the stroke, and it was also determined that the patient was at high thrombotic risk. Furthermore, given that PFO closure was already planned, the decision to apply the AtriClip was made.

Subsequent investigation into the cause of the patient’s significant thromboembolic events noted no notable hematologic history of hypercoagulable disorder, prior thromboembolic events, or structural abnormalities such as an atrial septal defect. Beta-2-glycoprotein 1 antibody screen was negative, silica clotting time screening-to-confirmatory (S/C) ratio was normal, and dilute Russell's viper venom time S/C ratio was negative. The patient was positive for anticardiolipin antibody during the hospitalization and was found to have an elevated IgM anticardiolipin and negative IgG anticardiolipin. Hypercoagulable workup by the hematology team was only notable for recommendations for age-appropriate malignancy screening and long-term monitoring for occult arrhythmias. CT of the abdomen and pelvis demonstrated no inferior vena cava thrombus nor concern for May-Thurner syndrome. The patient was initiated on warfarin for long-term anticoagulation, with an INR goal of 2.5-3.5 during the hospitalization. A higher goal was chosen initially, given that the patient developed multiple arterial and venous thrombi and was determined to be at a high thrombotic risk. During the hospitalization, the INR goal was decreased to 2-3, and he was discharged with this goal. He demonstrated continued clinical improvement and was ultimately discharged. On ambulatory follow-up, the patient was asymptomatic, without further clotting events. Two repeat measurements of anticardiolipin antibody were within normal limits, suggesting against a diagnosis of anticardiolipin antibody syndrome. The authors state that informed consent was not needed since this case report only describes de-identified information.

## Discussion

**Figure 5:**
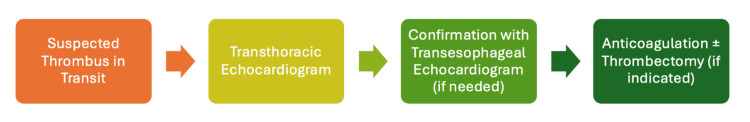
Flowchart that could be used when there is concern for a TIT. TIT: thrombus in transit.

We discuss a unique case of a patient with a PFO and TIT, who presented with multiple systemic thromboembolic events, including stroke, saddle PE, and deep vein thrombosis (DVT). TIT through a PFO carries a high risk for systemic embolization, particularly in the context of right-sided pressure elevation as seen with acute PE. In our case, TEE was instrumental in visualizing the mobile thrombus, which spanned approximately ten centimeters across the PFO. TEE was especially important as providers were not able clearly detect a PFO and were unable to accurately measure the size of the clot through the TTE images alone (Figure 5).

The DVT was determined to likely be the source of the patient’s stroke, given the presence of PFO and the location of the stroke. In patients with a PFO, there is limited literature on TIT, and most of the literature involving this unique finding involves patients with emboli in one or two vascular territories [3-6]. One case of PFO with thrombus in transit was reported in a patient with a DVT and saddle PE, but our case is unique, given our patient also had a cerebral artery occlusion [5]. Another case report described a patient with a PFO with thrombus in transit and PE who was treated with a thrombectomy and inferior vena cava filter placement, and our report is different, given the number of thrombi in our patient as well as the differences in management [3]. Our patient also received a broad imaging work-up to evaluate for thrombi burden. Our case is also distinct in presenting a patient with multi-organ embolization, highlighting the importance of PFO evaluation in patients with echocardiographic findings concerning for elevated right-sided pressures from acute PE. It remains critical for practitioners to investigate all potential locations where thrombi could be found in patients with TIT.

Management decisions in TIT are challenging due to the risk of paradoxical embolization, and options range from systemic anticoagulation to invasive surgical intervention. In this case, a key consideration was balancing the need for anticoagulation, preventing further embolization, with the risk of hemorrhagic transformation of the recent stroke. Although full-dose anticoagulation with heparin is typically avoided in acute stroke management due to the risk of hemorrhagic transformation, anticoagulation is crucial for patients with thrombus-in-transit to prevent further embolization [7]. Our patient presented with a left middle cerebral artery occlusion but was ineligible for tissue plasminogen activator (tPA) therapy, as he arrived outside the acceptable therapeutic time window, and thrombectomy was not an option due to the distal location of the occlusion. If the patient had presented within the appropriate time window for tPA, it could have been given, as the patient had no other clear contraindications for tPA. Consequently, anticoagulation was initiated to address the saddle PE, despite the increased risk of hemorrhagic conversion of the stroke.

Our patient was classified as intermediate-high risk for PE, given elevated troponin and BNP levels alongside evidence of right ventricular strain seen on TTE. Despite these findings, he remained hemodynamically stable with adequate oxygen saturation without supplemental support. One reason he likely remained hemodynamically stable was that full-dose anticoagulation with heparin was initiated after determining that the benefit of anticoagulation in the setting of PE outweighed the risk of hemorrhagic transformation of the left temporal infarct. Furthermore, thrombectomy may have mitigated right ventricle strain despite a large clot burden [8]. However, there was concern that the patient’s significant thromboembolic burden could lead to decompensation if it progressed. The recent stroke also rendered immediate cardiac surgery a high-risk option and further complicated management decisions. The patient was brought to the operating room two days after initial presentation for embolectomy given the TIT and the potential of recurrent systemic embolization. Our patient’s high thromboembolic burden and the presence of elevated IgM anticardiolipin antibodies initially raised concerns for antiphospholipid syndrome, although repeat testing was negative, and as a result, the patient does not meet one of the laboratory criteria for diagnosing antiphospholipid syndrome, which is the detection of IgM anticardiolipin antibodies at least 12 weeks apart. Furthermore, the patient was unable to transition to a direct oral anticoagulant due to insurance issues. Nevertheless, the possibility of a transient hypercoagulable state influenced the decision to pursue long-term anticoagulation with warfarin, given its proven efficacy in reducing thromboembolic recurrence in antiphospholipid syndrome patients [9,10].

## Conclusions

This case demonstrates that patients with intermediate- or high-risk PE, particularly those presenting with concurrent right- and left-sided thrombi (suggestive of paradoxical embolism) may benefit from a focused evaluation starting with TTE for patent foramen ovale and TIT followed by confirmation with TEE if TIT is suspected. Thorough anatomic evaluation of the patient’s stroke with subsequent CT and ultrasound imaging led to early recognition of TIT. A timely clinical recovery was achieved through admission to the cardiac intensive care unit, initiation of heparin, and operative management. Although the presentation of TIT is rare, early recognition of this complication may enable more accurate risk stratification and tailored management, potentially improving outcomes for patients with complex thromboembolic presentations.
